# Low postnatal serum IGF-I levels are associated with bronchopulmonary dysplasia (BPD)

**DOI:** 10.1111/j.1651-2227.2012.02826.x

**Published:** 2012-09-10

**Authors:** Chatarina Löfqvist, Gunnel Hellgren, Aimon Niklasson, Eva Engström, David Ley, Ingrid Hansen-Pupp, WINROP Consortium

**Affiliations:** 1 Department of Ophthalmology, Institute of Neuroscience and Physiology, Sahlgrenska Academy at University of GothenburgGothenburg, Sweden; 2 Department of Pediatrics, Institute of Clinical Sciences, Sahlgrenska Academy at University of GothenburgGothenburg, Sweden; 3 Division of Pediatrics, Department of Clinical Sciences Lund, Lund University HospitalLund, Sweden

**Keywords:** Bronchopulmonary dysplasia, Insulin-like growth factor-1, Premature infants

## Abstract

**Aim:**

To characterize postnatal changes in serum insulin-like growth factor-1 (IGF-I) in relation to development of bronchopulmonary dysplasia (BPD) in very preterm infants.

**Methods:**

Longitudinal study of 108 infants with mean (SD) gestational age (GA) 27.2 (2.2) weeks. Weekly serum samples of IGF-I were analysed from birth until postmenstrual age (PMA) 36 weeks. Multivariate models were developed to identify independent predictors of BPD.

**Results:**

Postnatal mean IGF-I levels at postnatal day (PND) 3–21 were lower in infants with BPD compared with infants with no BPD (16 vs. 26 μg/L, p < 0.001). Longitudinal postnatal change in IGF-I levels (IGF-I regression coefficient (β)), PNDs 3–21, was lower in infants with BPD compared with infants with no BPD (0.28 vs. 0.97, p = 0.002) and mean IGF-I during PMA 30–33 weeks was lower in infants with BPD as compared with infants without BPD (22 vs. 29 μg/L, p < 0.001). In a binomial multiple regression model, lower GA, male gender and lower mean serum IGF-I levels during PND 3–21 were the most predictive risk factors associated with BPD (r^2^ = 0.634, p < 0.001).

**Conclusion:**

Lower IGF-I concentrations during the first weeks after very preterm birth are associated with later development of BPD.

## Key notes

- Insulin-like growth factor-1 (IGF-I) plays a role in the developing foetal lung.
- Very preterm infants developing bronchopulmonary dysplasia (BPD) show lower postnatal IGF-I concentrations as compared with infants not developing BPD.
- Other risk factors for BPD development were low gestational age and male gender.

## Introduction

Postnatal growth failure is one of the most frequent consequences of extremely preterm birth (1) and poor postnatal growth contributes to the multifactorial aetiology of BPD (2). Insulin-like growth factors (IGFs) are important mediators of foetal growth and have also been associated with early postnatal growth following preterm birth (3,4).

Both pre- and postnatal inflammations have been described to be involved in lung injury and subsequent development of BPD (5,6). Further, impaired alveolarization in BPD has been associated with disruption of angiogenesis (7). The mechanisms that interfere with vascular growth are poorly understood. Of the many angiogenic factors, vascular endothelial growth factor (VEGF) has been shown to play a central role in pulmonary vascular development (7,8). Disrupted VEGF signalling reduces alveolarization and is involved in the development of BPD (9). In animal models of BPD, arrested vascular and alveolar development results in fewer and larger alveoli with decreased capillary density and nonsprouting, dysmorphic microvascular angiogenesis (7,10).

In the eye, IGF-I has been found to act as a permissive factor for the downstream signalling of VEGF through the mitogen-activated protein kinase (MAPK) and Akt pathway. In addition, IGF-I may increase VEGF protein levels by increasing the rate of VEGF mRNA transcription (11). Low levels of IGF-I that impair VEGF signalling are strongly associated with severe retinopathy of prematurity (ROP). In an animal model of ROP, IGF-I and one of its binding proteins, IGFBP-3, were shown to be important growth factors for normal retinal vascularization (11).

Recently, in adults with acute respiratory distress syndrome (ARDS), lower levels of circulating IGF-I and IGFBP-3 were independently associated with ARDS and with mortality (12). Data regarding circulating IGF-I and IGFBP-3 concentrations in premature babies who develop BPD are scarce. Increased expression of IGF-I and its receptor in postmortal lung tissue as well as increased concentrations of free IGF-I in tracheal aspirate fluid during the first postnatal week has been shown in preterm infants with BPD (13–15).

As impaired alveolar or microvascular development has been suggested in BPD (9,10), we wanted to extend our previous observations of the correlation between poor postnatal growth and subnormal growth factor levels, both experimentally and in infants, with abnormal angiogenesis and subsequent ROP (16) where we hypothesized that lower postnatal serum concentrations of IGF-I may be a risk factor associated with development of BPD.

## Patients and methods

### Study population

This is a retrospective study of two groups of infants who previously participated in a study on growth factors and growth, treated at two level III neonatal intensive care units in Sweden, Gothenburg and Lund (17,18) who both had longitudinal growth and growth factor levels collected into an on-line surveillance system called WINROP. The Gothenburg cohort consisted of 60 (22% born small for gestational age (SGA), 33% with BPD) infants born between December 1999 and April 2002 with gestational age (GA) at birth <32 weeks. The Lund cohort consisted of 48 (28% born SGA, 58% with BPD) infants born between January 2005 and May 2007 with GA at birth <31 weeks. The mean (SD) GA were 27.9 (2.2) and 26.4 (1.9) weeks for the Gothenburg and Lund cohort, respectively (p < 0.001). The Regional Ethical Review Boards of the Medical Faculties at Gothenburg and Lund University approved the study. The parents of the infants provided informed consent.

### Sampling of neonatal blood

Venous blood samples (0.5 mL) were obtained weekly and the serum stored at −80°C until assayed. Samples were taken when blood was drawn for other purposes from a patent arterial or venous line during the first postnatal weeks. Subsequent weekly blood samples were obtained by puncture of a peripheral vein.

### Clinical data

Antenatal data were obtained from maternal records. Relevant neonatal data were obtained prospectively from the infant’s records in which gender, GA at birth, birth weight (BW), Apgar score, patent ductus arteriosus (PDA), exposure to antenatal corticosteroids and postnatal corticosteroids, ventilator support, oxygenation requirement, BPD, necrotizing enterocolitis, intraventricular haemorrhage (IVH) and ROP were registered. Weight was recorded weekly and calculated into a SD score (using a growth reference curve adjusted for gender) (19). The infants in the study group were defined as SGA if the deviation in BW was more than 2 SD below that of the population mean adjusted for GA and gender (19).

### Quantitative analysis of IGFs

For the entire cohort, IGF-I and IGF-binding protein-3 (IGFBP-3) were analysed. IGF-II, IGFBP-1 and acid-labile subunit (ALS) were analysed only in the Gothenburg cohort owing to limited blood volumes in the Lund cohort.

IGF-I and IGFBP-3 concentrations were analysed using IGFBP-blocked radioimmunoassay (RIA) and a specific RIA, respectively, (Mediagnost GmbH, Tübingen, Germany). The measurement range for IGF-I at a dilution of 1:150 was 12-750 μg/L, which prompted us to dilute the IGF-I samples 1:50. As IGF-I levels in preterm infants are very low, the ability of the IGF-I assay to measure IGF-I levels in the low range was evaluated. Internal quality controls were developed by pooling serum from preterm infants, which was used throughout all assays to evaluate intra- and inter-assay coefficients of variation. All samples from one patient were analysed within one assay. IGF-I was measurable in all samples. The intra-assay coefficients of variation for IGF-I were 18%, 11% and 7% at 9, 33 and 179 μg/L, respectively. The inter-assay coefficients of variation for IGF-I were 29%, 11% and 6% at 9, 33 and 179 μg/L, respectively. The IGFBP-3 samples were diluted 1:300 and the intra-assay variation for IGFBP-3 was 10%, 7% and 6% at 716, 1750 and 3929 μg/L, respectively. The inter-assay coefficients of variation for IGFBP-3 were 21%, 12% and 11% at 725, 1859 and 3919 μg/L, respectively.

IGF-II concentrations were analysed using IGFBP-blocked RIA (Mediagnost GmbH). The IGF-II samples were diluted 1:101. All samples were analysed within the same assay. The intra-assay coefficients of variation for IGF-II were 10% and 8% at 260 and 821 μg/L, respectively.

IGFBP-1 concentrations were analysed with an enzyme immunoassay kit (Mediagnost GmbH). The IGFBP-1 samples were diluted 1:16. The intra-assay coefficients of variation for IGFBP-1 were 5% and 3% at 4 and 31 μg/L, respectively.

Total ALS concentrations were analysed with an enzyme immunoassay kit (DSL, Webster, Texas, United States). The ALS samples were diluted 1:100. The intra-assay coefficients of variation for ALS were 13% and 5% at 0.7 and 8 mg/L, respectively.

### BPD definition

Oxygen dependency at 28 days of postnatal age and at postmenstrual age (PMA) of 36 weeks was prospectively registered. In these cohorts, continuous need of supplemental oxygen at a PMA of 36 weeks was used as a definition of BPD (20).

### Statistical analysis

To assess whether clinical background variables differed between infants with and without BPD, Fisher’s exact test was used for categorical data, and either t-test or Mann–Whitney U-test was used for continuous data. All tests were two-sided with a 5% significance level. The odds ratio was used to describe the strength of a variable to predict development of BPD.

Multiple logistic regression analysis was used to analyse which variables that could be used to predict the development of BPD. Any variable with a p-value <0.2 in the univariate test was accepted as a candidate for the multivariate modelling along with variables known to be of clinical importance (See clinical variables in Table 1). When any two of the candidate variables were highly correlated, only one of the variables was included in the multiple regression model, to avoid problems with multicollinearity. The final multiple regression models were found using backward elimination. Statistical analyses were performed using the program package PAWS/SPSS version 17.0 (SPSS Inc., Chicago, IL, USA).

**Table 1:** Clinical characteristics [mean (SD) unless otherwise indicated] and univariate associations between pre- and postnatal variables and the development of bronchopulmonary dysplasia (BPD)

Clinical characteristic variable	No BPD (n = 50)	BPD (n = 58)	Odds ratio variable	BPD	
OR	95% CI
GA at birth (weeks)	28.7 (1.9)	25.9 (1.5)	GA at birth (weeks)	0.43	0.32–0.58***
BW (BW) (g)	1196 (341)	815 (210)	BW (50 g increment)	0.78	0.71–0.86***
BW SDS	−1.2 (1.3)	−1.1 (1.4)	BW SDS	1.05	0.79–1.39
Weight SDS 36 weeks	−1.8 (1.09)	−2.0 (1.1)	Weight SDS 36 weeks PMA	1.44	0.67–3.14
Apgar score 5 min med (range)	9 (5–10)	7 (2–10)	Apgar score 5 min	0.59	0.45–0.77***
Males, n (%)	20 (40)	36 (62)	Male gender	2.46	1.13–5.33**
SGA, n (%)	10 (20)	16 (28)	SGA	1.52	0.62–3.75
Preeclampsia, n (%)	14 (29)	9 (16)	Preeclampsia	0.47	0.18–1.20
PROM (>24h), n (%)	11 (20)	12 (21)	PROM (>24h)	0.92	0.36–2.32
NEC, n (%)	2 (4)	2 (3)	NEC	0.87	0.12–6.32
IVH, n (%)	2 (4)	11 (19)	IVH	5.62	1.18–26.71**
PDA, n (%)	8 (16)	24 (41)	PDA	3.71	1.49–9.29**
ROP treated, n (%)	2 (4)	10 (17)	ROP treatment	1.43	1.02–2.02**
Ventilator, n (%)	12 (24)	46 (100)	Ventilator	8.15	3.41–19.44***
Ventilator (days) med (range)	0 (0–17)	5 (0–100)	Ventilator (days)	1.17	1.08–1.28***
Antenatal Steroid n (%)	42 (84)	58 (100)	Antenatal steroid treatment	0.67	0.16–2.83
Postnatal steroids n (%)	4 (8)	24 (41)	Postnatal steroid treatment	8.12	2.57–25.58***

GA = gestational age; SGA = small for gestational age; PROM = premature rupture of membrane; NEC = necrotizing enterocolitis; IVH = intraventricular haemorrhage; PDA = patent ductus arteriosus; ROP = retinopathy of prematurity treated; BW, birth weight.

***p < 0.001, **p < 0.05.

## Results

### Risk factors for BPD

Increased odds ratios for developing BPD were found for the following risk factors: male gender, low GA, low BW, low Apgar score at 5 minutes, exposure to postnatal steroids, and occurrence of PDA, severe ROP and IVH (Table 1). The majority of infants in the study group were treated with supplemental oxygen. Compared with infants without BPD, infants with BPD spent significantly more days on a ventilator [median (range) 5 (0-100) vs. 0 (0-17) days] or on continuous positive airway pressure [46 (6–72) vs. 11 (0–50) days].

### Postnatal Serum IGF-I levels and development of BPD

**Figure 1:**
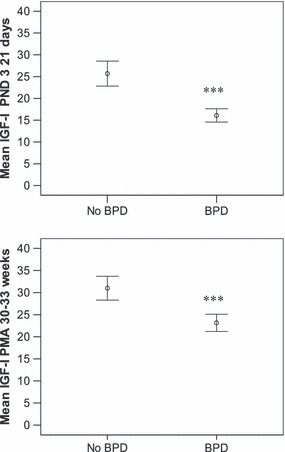
Mean serum IGF-I μg/L (95% CI) values for postnatal day (PND) 3–21 days (top) and PMA 30-33 weeks(bottom) in infants with and without bronchopulmonary dysplasia, ***p < 0.001.

To evaluate postnatal serum IGF-I concentration after birth, both mean IGF-I levels during postnatal day (PND) 3–21 and mean IGF-I levels during postmenstrual week 30–33 were calculated. Mean (SD) IGF-I levels PND 3–21 days were lower in infants with BPD compared with infants with no BPD (16 (5.80) vs. 26 (10.09) μg/L, p < 0.001). Further, the mean (SD) IGF-I levels at PMA 30-33 weeks were lower in infants with BPD than in those without BPD [23 (7.5) μg/L vs. 31 (9.6) μg/L, p < 0.001]. These differences remained significant after adjusting for GA (Fig. 1).

The mean IGF-I regression coefficient β (IGF-I-beta) between PND 3 and 21 and that for PND 22 and 44 was calculated in infants with BPD and in infants with no BPD. Postnatal change in IGF-I-β for PND 3–21 was lower in infants with BPD compared with infants with no BPD (0.28 vs. 0.97, p = 0.002). No difference was found for IGF-I-β for PND 22–44.

### Postnatal Serum IGF-II, IGFBP-1, IGFBP-3 and ALS and development of BPD

To evaluate postnatal serum IGF-II, IGFBP-1, IGFBP-3 and ALS concentrations after birth, mean levels during PMA 30–33 weeks were calculated. Mean IGF-II, IGFBP-1, IGFBP-3 and ALS serum levels at PMA 30–33 weeks were similar in infants with and without BPD (Table 2).

**Table 2:** Mean (SD) IGF-II, IGFBP-1, IGFBP-3 and ALS levels at postmenstrual age (PMA) 30-33 weeks are shown for infants with and without bronchopulmonary dysplasia (BPD).

Insulin-like growth factor binding proteins and ALS Mean (SD)	No BPD (n = 50)*	BPD (n = 58)*	p-Value
IGFBP-3 30–33 weeks (μg/L)	927 (253)	965 (233)	ns
IGF-II 30–33 weeks (μg/L)*	378 (64)	391 (78)	ns
IGFBP-1 30–33 weeks (μg/L)*	101 (62)	105 (140)	ns
ALS 30–33 weeks (mg/L)*	2.12 (0.90)	1.65 (0.84)	ns

For the entire cohort, IGFBP-3 were analysed.

IGF, Insulin-like growth factor; BP, binding protein; ALS, acid-labile subunit.

*IGF-II, IGFBP-1 and ALS were only analysed in the Gothenburg cohort. In the Gothenburg cohort: No BPD (n = 40); BPD (n = 20).

### Predictive risk factors for BPD in multivariate analysis

Multiple regression analyses were performed to assess the impact of pre- and postnatal factors available in the dataset. Two equivalent best fitted binomial multiple regression models were found. The first model included three independent variables: GA, gender and IGF-I as mean IGF-I at PND 3 and 21. This combination of variables explained between 48.0% (Cox and Snell R squared) and 64% (Nagelkerke R squared) of BPD development and correctly classified 84% of cases. The second model included three independent variables: GA, gender and mean IGF-I at PMA 30–33 weeks. This combination of variables explained between 49.0% (Cox and Snell R squared) and 64% (Nagelkerke R squared) of BPD development and correctly classified 84% of cases. These models showed that a boy with low GA and either a low mean IGF-I level at PND 3–21 or a low mean IGF-I level at PMA 30-33 weeks has the highest risk of developing BPD.

### Postnatal growth and BPD

There was no difference in BW or weight at PMA 36 weeks (SD scores) between infants with BPD and infants without BPD. Postnatal weight SD scores were lower in infants with BPD compared with infants without BPD at PMA 28-32 weeks (p < 0.001); however, this difference was not significant after adjusting for GA (Supporting Information Fig. S1).

## Discussion

In this study, we found that early low postnatal levels of circulating growth factor IGF-I predict an increased risk of BPD in preterm infants. In agreement with other studies, we found that lower GA and male gender independently predict an increased risk of BPD. This study also confirms that BW, exposure to postnatal corticosteroids, ROP and PDA are important factors associated with BPD. These same risk factors have been reported by a number of other studies from different countries.

To the best of our knowledge, the association between longitudinal IGF-I serum levels and BPD has not been studied previously. The findings of low postnatal serum IGF-I levels in infants with BPD are in agreement with earlier studies that show that the IGF system, particularly IGF-I itself, plays a key role in the mediation of growth in early infancy.

The activities of IGF-I are regulated by its binding proteins. The pattern of IGF regulation changes profoundly during the perinatal period; IGFBP-1 and IGFBP-2 dominate in the intra-uterine environment, whereas IGFBP-3 is the major serum IGFBP later in life. A delayed decrease in the foetal IGFBPs, such as the increase in IGFBP-1 observed after glucocorticoid therapy (21), could theoretically lower free IGF-I and contribute to reduced growth catch-up. In a subgroup of this study population, we found no difference in the longitudinal development of serum IGFBP-1 between infants with BPD and infants without BPD. We also did not find any significant differences in IGF-II or ALS serum levels between infants with BPD and without BPD. These results were obtained in a subgroup; the Gothenburg cohort of 60 infants who had longitudinal weekly blood samples available in 20 infants developing BPD and in 40 infants not developing BPD. The infants in the Lund cohort were significantly more premature at birth than the Gothenburg cohort and therefore both GA and NICU sites were evaluated in the analysis. We found a tendency for ALS levels at PMA 30-33 to be lower in infants with BPD. ALS is undetectable in foetal serum at 27 weeks of gestation, and in this study the infants with BPD were born at a lower GA, which could be an explanation for the lower levels of ALS seen in infants with BPD. The serum levels of ALS measured at PMA 30-33 weeks in infants born very or extremely preterm correspond to levels previously reported at birth for preterm infants born between GA 26 and 36 weeks (22).

Circulating IGF-I concentrations do not necessarily reflect local production in tissues. The cells responsible for the synthesis of IGF-I in the lung are type II pneumocytes, alveolar macrophages and mesenchymal cells (14,15). Recent studies have shown that IGF-I mRNA expression in the lung is predominant during foetal life and decreases before birth, becoming barely detectable in the neonatal lung. However, in response to injury, alveolar epithelial cells and inflammatory cells release and activate a number of cytokines and growth factors involved in fibroblast migration and proliferation, and change to myofibroblasts, leading to accumulation and remodelling of the extracellular matrix (23). One of these factors is IGF-I, which enhances the proliferation of human foetal lung fibroblasts and stimulates collagen production during lung injury, with an important role in the lung injury/repair process. In line with this, increased concentrations of free IGF-I have been observed in early postnatal tracheal aspirate fluid from intubated preterm infants who later develop BPD (13).

The cause of growth failure in extremely preterm infants who develop significant BPD remains unclear. It is well known that BPD is associated with poor growth, which has mainly been attributed to malnutrition and increased energy expenditure. However, recent studies have shown that if recommended intakes are received, preterm infants do not develop cumulative energy and protein deficits (24). We speculate that the low IGF-I levels at PMA 30–33 for infants who developed BPD are a consequence of a suboptimal milieu for an immature infant during the critical first weeks of postnatal life. Poor postnatal growth and subsequently low IGF-I levels are strongly influenced by nutrition, severe infection, acidosis and other metabolic disturbances (3,4). In our study, no significant differences in nutritional intake according to presence or absence of BPD were observed in any of the cohorts (data not shown).

We also found that boys were more likely to have BPD than girls. This is in line with earlier studies reporting that male gender is associated with an increased risk for BPD. Why male infants are at a greater risk of developing lung disease is unclear. Antenatal steroids are given to promote lung maturation and improve outcome. Female preterm infants have been found to have less oxidative stress, increased antioxidant activity and better clinical outcomes than males, independent of antenatal steroid use (25). In addition, girls exhibit a larger reduction of BPD than boys with avoidance of hyperoxia (26). Preterm male infants have been shown to need more initial respiratory and circulatory support than female infants (27). Deficiency of Clara cell 10 kDa protein (CC10), the major secretory protein of Clara cells thought to play a protective role in the lung owing to its anti-inflammatory properties, is associated with high expression of cytokines in the lung. Ramsay et al. showed that a lower level of CC10 is associated with an increased risk of BPD (28). Interestingly, in an animal model of BPD, replacement of rhIGF-I increased the number of Clara cells (29). In addition, IGF-I-treatment in chronically hypoxic rats resulted in more weight gain compared with vehicle-treated rats. These results suggested that IGF-I treatment promotes anabolism under chronic hypoxic conditions in which caloric intake is decreased (30).

In conclusion, our data demonstrate that low early postnatal serum IGF-I levels are associated with development of BPD. Future studies should be designed to validate the present findings to verify postnatal IGF-I as a biomarker for BPD development and to further the understanding of IGF-I in postnatal lung development.

## Statement of financial support

This study was supported by the Swedish Medical Research Council (grant # 2008-2842, 14940), Government grants (#ALFGB-21611), VINNOVA (grant # 2009-01152, 2009-00221), the Torsten and Ragnar Söderberg Foundation, the Skåne Council Foundation for Research and Development, the Linnéa and Josef Carlsson Foundation, and the NEI EY017017, EY022275 RPB Sr. Invest., P01HD18655 (LEHS). The application to prevent retinopathy of prematurity (ROP) by administering IGF-I is covered by patents and patent applications owned by Children′s Medical Center Corporation, Boston, United States, and Premacure AB, Uppsala, Sweden. Five of the authors (C.L., I.H.P., A.L.H., D.L. and A.H.) own shares in a company controlling Premacure AB. The remaining authors have nothing to declare.

## Notes

Figure S1 Mean weight SD score (95% CI) values in relation to PMA for infants with BPD (dotted line) and infants without BPD (solid line).
