# Supplementary material for: Low postnatal serum IGF-I levels are associated with bronchopulmonary dysplasia (BPD)
Source: Acta Paediatr. 2012 Sep 10;101(12):1211–6. doi: 10.1111/j.1651-2227.2012.02826.x (PMC3569611; doi:10.1111/j.1651-2227.2012.02826.x)
Supplement: Figure S1 — Mean weight SD score (95% CI) values in relation to PMA for infants with BPD (dotted line) and infants without BPD (solid line). [file apa0101-1211-SD1.doc]

**Figure S1.** Mean weight SD score (95% CI) values in relation to PMA for infants with BPD (dotted line) and infants without BPD (solid line). Infant with BDP have lower weight than infants without BPD around PMA 30 weeks. ***p<0.001.

-------------***-------------
